# Stem Cells in Translational Cancer Research

**DOI:** 10.1155/2015/281072

**Published:** 2015-05-11

**Authors:** Oswaldo Keith Okamoto, Ander Matheu, Luca Magnani

**Affiliations:** ^1^Department of Genetics and Evolutionary Biology, Human Genome and Stem Cell Research Center, Biosciences Institute, University of São Paulo, 05508-090 São Paulo, SP, Brazil; ^2^Neurooncology Group, Department of Oncology, Biodonostia Institute, Paseo Dr. Beguiristain s/n, 20014 San Sebastián, Spain; ^3^Department of Surgery and Cancer, Imperial Centre for Translational and Experimental Medicine, Imperial College, Hammersmith, London W12 1NN, UK

Since the seminal studies by Fialkow et al. in the seventies [1] and by Bonnet and Dick in the nineties [2], where an organized tumor cell hierarchy originating from transformed hematopoietic stem cells was found in myeloid leukemias, the cancer stem cell (CSC) model of tumor development drew the attention of the scientific community and became the focus of extensive discussion. This theme gained momentum over the past decade, with the identification of stemlike cells as drivers of tumor initiation, recurrence, and metastasis spread in a variety of nonhematopoietic human cancers. These characteristics postulated them as novel and promising therapeutic targets for cancer, especially in the context of drug-resistant tumors [3].

The origin of these stemlike cells is being extensively studied. In some types of tumors, such as in blood, brain, colon, and skin cancer, it has been shown that adult stem cells are prone to transformation due to a longer half-life within tissues compared to their cell progenies, facilitating the accumulation of oncogenic mutations as a result of prolonged exposure to genotoxic stresses [4]. In other cases,* de novo* acquisition of stem cell properties may occur in either normal or neoplastic cells due to genetic/epigenetic aberrations [5]. Finally, the CSC model has been put forward to explain why tumors like breast cancer might return years after surgery. In this situation, lower proliferation rate might allow cells to escape from aggressive therapy and allow for genetic/epigenetic evolution.

Advances in the field, however, point to varied and more complex models of tumor development than previously envisioned, in which the stemlike phenotype may also be dynamically acquired by cancer cells through interaction with stromal cells and soluble factors [6]. A relevant question timely addressed in this special issue is the understanding of the contribution of resident stem cells to the tumor microenvironment (TME). This is of major relevance since they may significantly influence tumor progression, aggressiveness, and response to therapy. The contribution of pericytes to tumor growth, angiogenesis, metastasis, and evasion of immune destruction, which are classic hallmarks of cancer, is comprehensively reviewed by A. L. Ribeiro and O. K. Okamoto. In their article, the authors also discuss a pericyte-mediated regulation of stemness properties in cancer cells and argue in favor of pericytes as cellular targets for new cancer therapies aiming at the TME. Indeed, many new anticancer therapies targeting the TME are under development, with currently approved antiangiogenic drugs as examples of such strategy. However, events of tumor recurrence and poor response to antiangiogenic therapy still puzzle researchers and emphasize the need for continuous studies. In the article by M. Marçola and C. E. Rodrigues, the involvement of endothelial progenitor cells in tumor angiogenesis is critically reviewed. In their article, the specific roles of different members of the VEGF family of growth factors, as well as the relevance of the vascular niche to stemness in cancer cells, are also carefully discussed.

Another practical issue of clinical relevance is the rigorous evaluation of potential oncogenic risks associated with stem cell therapy. Long-term safety issues must be properly addressed before stem cell-based therapies enter clinical trials, but such studies are outnumbered in the literature by studies evaluating therapeutic effects based on restoration of tissue integrity and physiological balance. While the original article by T. Jazedje et al. addresses this issue at the preclinical stage, showing in a murine breast adenocarcinoma model that mesenchymal stromal cells (MSC) may exert either pro- or antitumorigenic effects depending on the experimental condition, R. Schweizer et al. bring a more clinical perspective to this issue, presenting an interesting discussion about the possible oncogenic hazards of adipose-derived MSC in breast cancer patients subjected to breast reconstruction after mastectomy.

Finally, due to their tumor homing properties, the use of stem cells as vehicles to deliver suicide genes is a strategy highly pursued in cancer gene therapy. However, low delivery efficiency and off-target effects are common limitations of current expression systems. In the original article by Y. Luo et al., the authors elegantly characterize a novel inducible transgene expression system based on the action of neural stem cells that could be further explored to treat malignant gliomas.

In summary, the articles compiled in this special issue highlight how the growing investigation of tumor development through the lens of stem cell biologists is significantly impacting basic and translational cancer research. This interplay between stem cell and tumor biology offers an exceptional opportunity to improve our knowledge about cancer, one of the leading causes of death worldwide. Advances in this field are expected to bring significant impacts in cancer diagnosis and therapy.

*Oswaldo  Keith  Okamoto* *Oswaldo  Keith  Okamoto* *Ander  Matheu* *Ander  Matheu* *Luca  Magnani* *Luca  Magnani*
